# Solution-Processed OLEDs Based on Thermally Activated Delayed Fluorescence Copper(I) Complexes with Intraligand Charge-Transfer Excited State

**DOI:** 10.3390/molecules26041125

**Published:** 2021-02-20

**Authors:** Teng Teng, Jinfan Xiong, Gang Cheng, Changjiang Zhou, Xialei Lv, Kai Li

**Affiliations:** 1 College of Materials Science and Engineering, Shenzhen University, Shenzhen 518055, China; tteng-happy@outlook.com (T.T.); xiongjinfan123@gmail.com (J.X.); changjiang7pao@sina.com (C.Z.); lvxl0907@163.com (X.L.); 2 College of Physics and Optoelectronic Engineering, Shenzhen University, Shenzhen 518060, China; 3 State Key Laboratory of Synthetic Chemistry, HKU-CAS Joint Laboratory on New Materials, Department of Chemistry, The University of Hong Kong, Pokfulam Road, Hong Kong, China; ggcheng@hku.hk

**Keywords:** Cu(I) complexes, TADF, intraligand charge-transfer, OLED, solution process

## Abstract

A new series of tetrahedral heteroleptic copper(I) complexes exhibiting efficient thermally-activated delayed fluorescence (TADF) in green to orange electromagnetic spectral regions has been developed by using D-A type N^N ligand and P^P ligands. Their structures, electrochemical, photophysical, and electroluminescence properties have been characterized. The complexes exhibit high photoluminescence quantum yields (PLQYs) of up to 0.71 at room temperature in doped film and the lifetimes are in a wide range of 4.3–24.1 μs. Density functional theory (DFT) calculations on the complexes reveal the lowest-lying intraligand charge-transfer excited states that are localized on the N^N ligands. Solution-processed organic light emitting diodes (OLEDs) based on one of the new emitters show a maximum external quantum efficiency (EQE) of 7.96%.

## 1. Introduction

Emissive triplet states of transition metal complexes underpin a wide range of advanced optoelectronic applications, such as organic light emitting diodes (OLEDs) and biological studies [1,2,3,4,5,6,7]. The relatively long excited-state lifetimes in the microsecond regime owing to the spin-forbidden nature of triplet excited states are also desired for photochemical reactions and triplet-triplet annihilation based upconversion (TTA-UC) involving bimolecular electron and/or energy transfer processes [8,9,10]. These fields have been dominated by phosphorescent transition metal complexes based on the platinum group metals (PGMs), such as iridium, platinum, and ruthenium because of their significant spin–orbit coupling (SOC) constants [1,2,3,4,5,6,7,8,9,10]. The practical use of Ir(III) emitters in current commercial OLEDs possibly suggests the advantage of metal complexes in the device operational stability. However, the natural abundances of these metals are extremely low, thereby limiting their practical applications. Therefore, research endeavors to the exploration of alternative luminescent molecular materials with reliable resources and low cost is demanding [11,12]. In this context, luminescent coinage metal complexes have been studied for decades but their applications to OLEDs are greatly restricted. The relatively weak SOC constants (for Ag and Cu) or the high oxidation potentials (for Au(III)) of the coinage metal ions renders their triplet excited states to have long emission lifetimes that are detrimental to electroluminescence devices [13,14].

**Figure 1:**
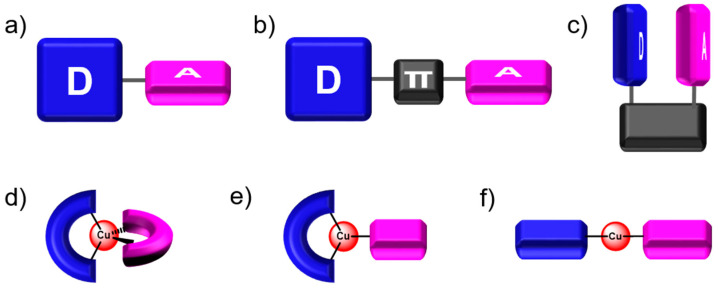
Molecule designs of organic and Cu(I) thermally-activated delayed fluorescence (TADF) emitters: (**a**) highly twisted D-A structure with through-bond charge transfer (TBCT) excited state; (**b**) separated D and A segments by a π spacer; (**c**) intramolecular D-A pair with through-space charge transfer (TSCT) excited state; (**d**) tetrahedral, (**e**) trigonal, and (**f**) linear coordination geometries for Cu(I) complexes with predominant ligand-to-ligand charge transfer (LLCT) excited state.

The thermally activated delayed fluorescence (TADF) of pure organic compounds has clearly shown the capability to harvest triplet excitons in OLEDs in the absence of heavy atoms [15]. Instead of radiative decay to ground state, the triplet excited state of a TADF emitter is thermally pumped to the singlet excited state through reverse intersystem crossing (RISC) which then radiates rapidly. In parallel with the advances of pure organic luminescent materials, TADF cuprous complexes have also witnessed a great stride which now show promising device performances that become comparable to phosphorescent noble metal complexes in terms of device efficiency [16,17,18,19,20,21,22,23,24,25,26,27]. The TADF mechanism allows Cu(I) complexes to bypass the slow radiative decay of triplet excited states of Cu(I) emitters. In addition, the SOC of light metals, although weak, may still has non-negligible positive effect in boosting the RISC process. In comparison with the pure organic TADF emitters, which are designed by engineering, the donor, and acceptor through a highly twisted through-bond connection or a through-space charge-transfer interaction (Figure 1a–c), molecular geometries of Cu(I) complexes can be facilely controlled by the design or selection of ligand(s). Representative TADF Cu(I) complexes with tetrahedral, trigonal, and linear coordination geometries are shown in Figure 1 (Figure 1d–f). Most of the TADF Cu(I) complexes adopt donor-metal-acceptor configuration and, thus, have ligand-to-ligand charge transfer (LLCT) excited states. In the literature, it has been shown that the TADF can also be achieved from intraligand charge-transfer excited state in Pd(II), W(VI), and Au(III) complexes supported by tetradentate ligands [28,29,30]. Likewise, this concept has recently been demonstrated on Cu(I) and Ag(I) complexes using simple TADF-inactive donor-acceptor type ligands (Figure 1f) [19,31,32]. The coordination with metal ion was found to affect the relative energy levels of lowest lying singlet and triplet excited states of the ligand, thus switching on the ligand-based TADF.

**Scheme 1:**
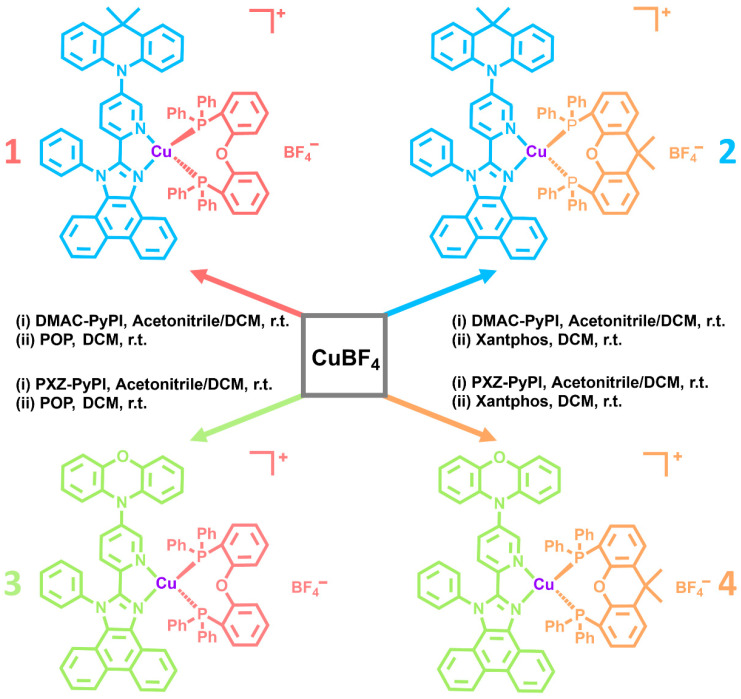
Synthesis and chemical structures of the Cu(I) complexes **1**–**4** in this work.

Herein, four new Cu(I) complexes (Scheme 1) exhibiting intraligand TADF have been developed. Pyridine substituted 1-phenyl-1*H*-phenanthro[9,10-d]imidazole (PyPI) was modified with strong electron-donors of dimethyl-9,10-dihydroacridine (DMAC) or 10*H*-phenoxazine (PXZ) to construct charge-transfer type ligand. The bis[2-(diphenylphosphino)phenyl]-ether (POP) and 4,5-bis(diphenylphosphino)-9,9-dimethylxan-thene (Xantphos) ligands were used as the auxiliary ligands. These complexes exhibit efficient TADF with photoluminescence quantum yields (PLQYs) of up to 0.71 in doped film. Dependent on the donor, the delayed fluorescence lifetimes vary appreciably from 4 μs to 21 μs. The involvement of triplet locally excited (^3^LE) state in the RISC process likely exerts a positive effect on the RISC rate. Solution-processed OLEDs based on this type of emitter have been fabricated and achieved maximum external quantum efficiency (EQE) of up to 7.96%.

## 2. Results and Discussion

### 2.1. Synthesis and Structures

The syntheses of the diimine ligands, 2-(5-(9,9-dimethylacridin-10(9*H*)-yl)pyridin-2-yl)-1-phenyl-1*H*-phenanthro[9,10-d]imidazole (**DMAC-PyPI**) and 10-(6-(1-phenyl-1*H*-phenanthro[9,10-d]imidazol-2-yl)pyridin-3-yl)-10*H*-phenoxazine (**PXZ-PyPI**), have been described elsewhere [31]. As depicted in Scheme 1, all four cuprous complexes were prepared using a typical method and obtained as crystals in high yields of 77%–84%. All complexes have been characterized by ^1^H and ^31^P{^1^H} NMR spectra and elemental analysis. The thermal stability of all the complexes was examined by thermogravimetric analysis (TGA) under argon atmosphere. Their decomposition temperatures (T_d_) at a weight loss of 5% were determined to be from 345 °C to 381 °C (Figure S1, Supplementary Materials).

**Figure 2:**
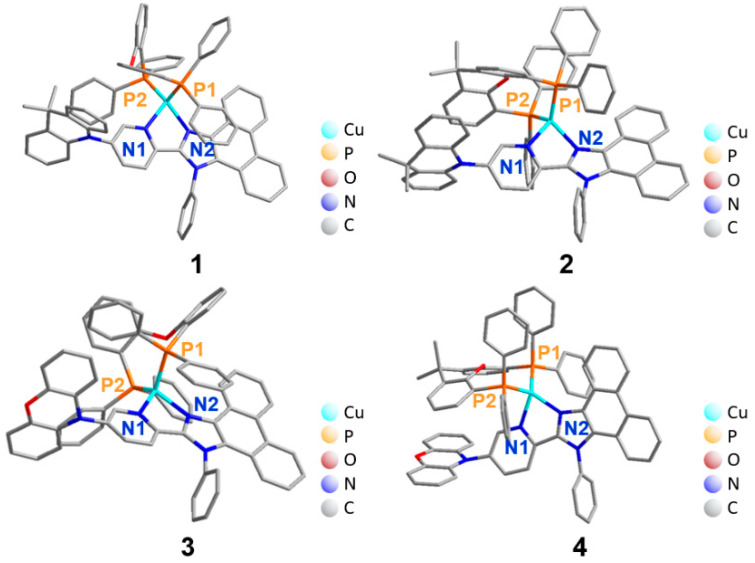
Crystal structures of complexes **1**–**4**.

Molecular structures of **1**–**4** have been determined through single-crystal X-ray diffraction studies and the crystal data are provided in Table S1 (Supplementary Materials). The selected bond lengths of Cu-N and Cu-P bonds and bond angles around the metal center are compiled in Table S2. As depicted in Figure 2, the Cu(I) ions in all crystal structures bind to a N^N ligand and a P^P ligand in a distorted tetrahedral coordination geometry. The Cu-N and Cu-P bond lengths of 2.056(3)–2.139(3) Å and 2.218(2)–2.2941(15) Å, respectively, are in the range for cationic [Cu(N^N)(P^P)]^+^ complexes in the literature [33]. Likewise, the N-Cu-N and P-Cu-P bond angles in the range of 78.10(16)–79.98(11) and 115.41(4)–118.20(8), respectively, are among the typically reported values. The dihedral angles defined by the N^Cu^N and P^Cu^P planes are 85.3–89.8°, revealing nearly orthogonal orientation of the chromophore N^N and auxiliary P^P ligands (Table S2, Supplementary Materials). The PXZ moieties in **3** and **4** are planar and strongly twisted from the N-Cu-N coordination plane by ca. 68° and 69°, respectively. In contrast, the DMAC in **1** and **2** slightly deviate from an ideal plane.

### 2.2. Photophysical Properties

**Figure 3:**
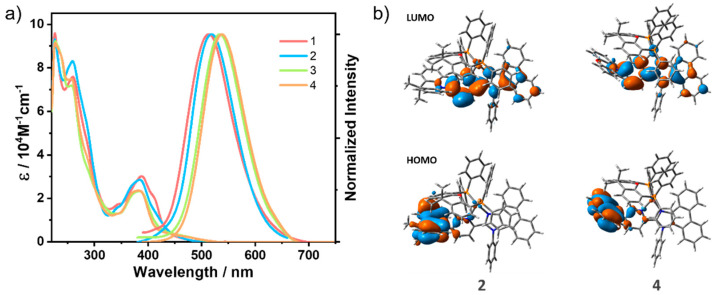
(**a**) UV-vis absorption and emission spectra of **1**–**4** in CH_2_Cl_2_ solution (1 × 10^−5^ M) at 298 K; (**b**) Frontier molecular orbitals for **2** and **4** at their optimized ground state geometries.

Figure 3a shows the absorption spectra of the Cu(I) complexes in CH_2_Cl_2_ at room temperature. The intense absorptions at λ < 300 nm are composed of π→π* transitions of the phenyl-1*H*-phenanthro[9,10-d]imidazole (PI), the donor and the phosphine segments. The absorptions with maxima at 380–388 nm with molar absorptivity of ca. 3 × 10^4^ M^−1^ cm^−1^ are tentatively assigned to intraligand π→π* transition of the planar PyPI moiety, probably with an admixture of charge transfer transition from the Cu/P^P to the N^N ligand. The weaker and broad absorption tailing to 500 nm for **1**/**2** and to 530 nm for **3**/**4** are assigned as intraligand charge-transfer (ILCT) transitions from the donor (DMAC and PXZ) to the PyPI; the redshifted absorptions for **3**/**4** in comparison to **1**/**2** are consistent with the stronger electron-donating capability of PXZ. The assignment of the lowest excited singlet state is supported by the density functional theory (DFT) calculations. As depicted in Figure 3b and Figure S2 (Supplementary Materials), the HOMOs in all complexes predominantly localize on the donor (PXZ or DMAC) and the LUMOs are distributed over the PyPI moiety. Previously, similar ILCT character of the lowest excited states have been shown for Ag(I) complexes based on ligands **DMAC-PyPI** and **PXZ-PyPI** [31].

Emission properties of **1**–**4** were firstly examined in CH_2_Cl_2_ at room temperature. As depicted in Figure 3a, these complexes exhibit broad, structureless emissions with emission maxima of 514–537 nm upon photo-excitation (λ_ex_ = 365 nm). The almost same emission energy and similar spectral profile for **1**/**2** and for **3**/**4** are in line with the N^N ligand-dominated excited state. The significant overlap between the lowest energy absorption and the emission spectra reveals the emission origin from the first singlet excited state (S_1_). The PLQYs of **1**–**4** in deaerated CH_2_Cl_2_ are 0.042–0.095. The excited state structural flattening and thus exciplex formation are typically encountered for Cu(I) complexes with metal-to-ligand charge-transfer (MLCT) excited state, which result in low photoluminescence quantum yields [33,34]. In view of the sterically congested features of the P^P and N^N ligands and the ligand-dominated excited state, such structural change is unlikely to occur in the present cases. Instead, torsional dynamics of the donor moieties and the phenyl ring on the N^N ligand in fluid solution are conceived to induce fast non-radiative decay, thus giving low emission efficiencies of **1**–**4**.

**Figure 4:**
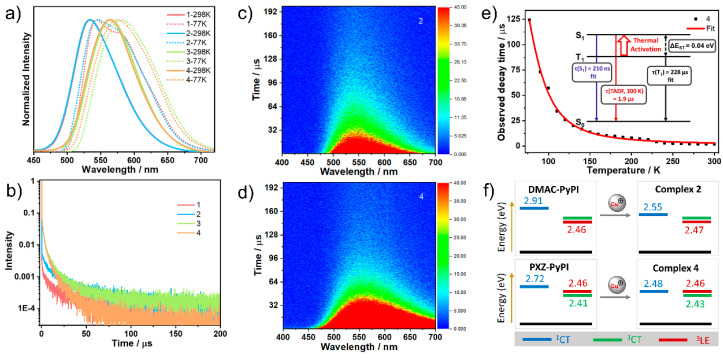
Photophysical properties of the Cu(I) complexes in DPEPO films: (**a**) photoluminescence (PL) spectra at 298 K and 77 K; (**b**) transient PL characteristics for **1**–**4**; time-resolve PL spectra of (**c**) **2** and (**d**) **4**; (**e**) analysis of the variable temperature emission lifetimes of **4** using a Boltzmann-type equation; (**f**) proposed excited state energy levels for **2** and **4**.

Then, the emission properties of **1**–**4** in bis[2-(diphenylphosphino)phenyl]ether oxide (DPEPO), 1,3-bis(N-carbazolyl) benzene (mCP), and poly-(methyl methacrylate) (PMMA) films were studied. All the numerical data are compiled in Table 1 and Table S3 (Supplementary Materials). As depicted in Figure 4a, the photoluminescence (PL) spectra (λ_max_ = 533–565 nm) of all the complexes in DPEPO films (10 wt%) at room temperature show characteristic CT emissions that are redshifted from their emissions in CH_2_Cl_2_ by 15–31 nm; the larger bathochromic shift for **3** and **4** than for **1** and **2** are likely due to the stronger charge-transfer excited state nature of the former two complexes. This can be interpreted by the stronger electron-donating capability of PXZ compared with DMAC. Remarkably, the PLQYs for all complexes are increased to 0.42–0.71 in the DPEPO films (Table 1) which can be ascribed to the suppressed intramolecular motions in the rigid solid matrix. Transient photoluminescence decay studies of the doped DPEPO films registered at their emission maxima show dominant decay times of 4.3–24.1 μs under argon atmosphere (Figure 4b). As shown in Figure 4c,d, similar excited state dynamics were also supported by the time-resolved emission mapping measurements of **2** and **4**. The emission lifetime scale precludes the phosphorescence nature for their emissions because triplet excited states featuring very minor d orbital participation of Cu(I) are presumed to have much longer emission lifetimes. This speculation can be manifested by the much longer emission lifetime of **4** at lower temperature (vide infra). The strong temperature independence of emission lifetimes reveals the large variation of radiative decay rate with temperature changes, which is determined by the emitting state nature. Thus, the emissions for **1**–**4** at room temperature are assigned to TADF, as that for Ag(I) analogues with the same ligands [31]. Notably, prompt fluorescence was not observed in the transient photoluminescent studies of each complex. The absence of prompt fluorescence in TADF metal complexes have been frequently found for Cu(I), Au(I), Au(III) complexes, which can be ascribed to the trivial but non-negligible heavy atom effect of the metal center in boosting the ISC process. To confirm the emission mechanism, phosphorescence spectra of the four emitters were probed at 77 K. As depicted in Figure 4a, complexes **1** and **2** exhibit vibronically structured emission with peak/shoulder at 544 nm/581 nm and 545 nm/579 nm, respectively. Differently, complexes **3** and **4** exhibit broad profiles with emission peaks at 582 nm and 575 nm, respectively. The change in the emission band profile and the cryogenic shift are typical for TADF molecules, which lead to the conclusion that the triplet excited states at 77 K have significant ^3^LE and ^3^ILCT characters for **1**/**2** and **3**/**4**, respectively. The ∆*E*_ST_ values for all the complexes are estimated to be 0.05–0.11 eV based on the emission onsets of their fluorescence and phosphorescence spectra (Table 1), favorable for the TADF emissions at room temperature. For comparison, the emission properties of **1**–**4** in the films of PMMA and mCP were also studied. As depicted in Figures S3 and S4 and Table S3 (Supplementary Materials), similar emission spectral characteristics to those in DPEPO at both room temperature and 77 K were observed for all the complexes.

Variable temperature emission lifetimes of **4** in its powder state were measured at 77–300 K to further confirm the emission mechanism. According to the literature report [35], the observed decay time (τ_obs_) can be described by the following equation in the tested temperature range:

$$\tau \;=\;\frac{1+\frac{1}{3}\exp\left(-\frac{\Delta E_{\mathrm{ST}}}{k_{B}T}\right)}{\frac{1}{\tau \left(T_{1}\right)}+\frac{1}{3\tau \left(S_{1}\right)}\exp\left(-\frac{\Delta E_{\mathrm{ST}}}{k_{B}T}\right)}$$

where k_B_ is the Boltzmann constant, τ(S_1_) and τ(T_1_) are the decay lifetimes of the S_1_ and T_1_ states, and Δ*E*_ST_ represents the energy difference between the S_1_ state and T_1_ state.

The experimental data were found to agree well with Boltzmann-type equation (Figure 4e). At low temperature (77 K), the observed lifetime of 124 μs indicates that the cuprous complexes emit exclusive phosphorescence. As the temperature increases from 77 K to 130 K, the emission lifetime shortens quickly as a result of thermal population of S_1_, which equilibrates with the T_1_ state according to Boltzmann statics. Further increasing the temperature to 240 K leads to a steady decrease of the lifetime. In this temperature range, the emission is composed of radiations from both S_1_ and T_1_ states; that is, TADF and phosphorescence. At the temperatures above 240 K, the lifetimes keep almost unchanged, suggesting that the population of S_1_ is highly favored and TADF dominates the emission. The Δ*E*_ST_ is fitted to be 0.04 eV, which is in good agreement with the estimated value (0.05 eV) based on the emission spectra. The similar ∆*E*_ST_ values estimated using both methods reveal the similar molecular geometries at both room temperature and 77 K [36].

A notable finding in the photophysical properties of this series of Cu(I) complexes is the large difference of the delayed fluorescence lifetimes between **1**/**2** and **3**/**4**. The 4–6 fold shorter lifetimes of **3**/**4** obviously reflect their faster RISC rate. A schematic diagram showing the effect of copper ion coordination on the excited states energy levels of **DMAC-PyPI** and **PXZ-PyPI** and on the excited state electronic structures is shown in Figure 4f. The S_1_ and T_1_ energies of **DMAC-PyPI** and **PXZ-PyPI** have been studied elsewhere and the large Δ*E*_ST_ values of 0.31–0.45 eV rationalize their prompt fluorescence [32]. It is noted that the T_1_ energies of both ligands are close to each other with a small difference of only 0.05 eV despite the relatively larger difference of ca. 0.2 eV for their S_1_ states. Further, the phosphorescence spectra of **DMAC-PyPI** is characterized by a vibronic progression while that of **PXZ-PyPI** is relatively broad and structureless. Therefore, a significant admixture of ^3^LE character of the lowest triplet excited states is proposed for both ligands but energetic orderings of the ^3^LE and ^3^CT are reversed (Figure 4f). Strikingly, coordination to Cu(I) ion does not change the triplet excited state energy level and the emission spectral profile. As shown in Figure 4a, the T_1_ states of **1** and **2** have larger ^3^LE parentage while that of **3** and **4** are dominated by the ^3^CT state. Instead, the ^1^CT states are stabilized by 0.26–0.36 eV upon metal coordination, leading to a decrease of Δ*E*_ST_ that becomes thermally accessible for TADF. In particular, the ^1^CT and ^3^LE for **3** and **4** are estimated to be energetic degenerate which is highly beneficial for RISC.

### 2.3. Electroluminescence

**Figure 5:**
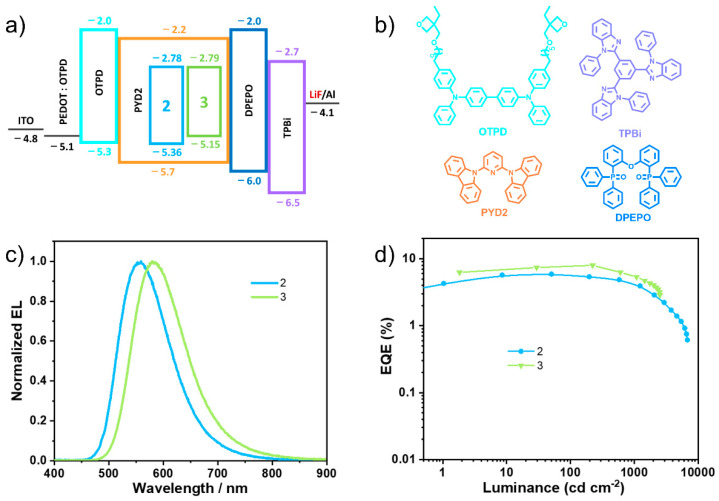
(**a**) The device configuration and (**b**) chemical structures of the organic materials used in the devices; (**c**) electroluminescence (EL) spectra of the optimized devices; (**d**) plots of external quantum efficiency versus luminance.

To examine the potential of this series of complexes for electroluminescence (EL), complexes **2** and **3** were selected for device fabrication. Complex **2** was studied because of the highest PLQY among the four complexes. Since the emission lifetime is also a crucial factor in determining the device performances, complex **3** was studied for comparison in view of its moderate PLQY but a beneficial short emission lifetime. The device structure of ITO/PEDOT:PSS (50 nm)/OTPD (4 nm)/PYD2: emitter (60 nm)/DPEPO (10 nm)/TPBi (40 nm)/LiF (1.2 nm)/Al (100 nm) was designed according to that for similar Ag(I) complexes [31]. The energy level diagrams and chemical structures of the organic materials are shown in Figure 5a,b. The HOMO levels of the Cu(I) complexes were determined by electrochemical studies and the LUMO levels can be deduced in combination with the optical bandgaps (Figure S5 and Table S4). For example, the HOMO/LUMO levels are estimated to be −5.36/−2.78 eV for **2**. Di(9*H*-carbazol-9-yl)pyridine (PYD2) with a good hole-transporting ability and high triplet energy level (3.03 eV) was used as host material in the emissive layer (EML). DPEPO and 1,3,5-tris(1-phenyl-1*H*-benzo[d]imidazol-2-yl)benzene (TPBi) function as hole/exciton blocking and electron transporting materials, respectively. The cross-linkable *N*,*N*′-bis(4-(6-((3-ethyloxetan-3-yl)methoxy))-hexylphenyl)-*N*,*N*′-diphenyl-4,4′-diamine (OTPD) was used to facilitate the hole injection into the emissive layer (EML) and prevents exciton quenching from the PEDOT:PSS layer. The EML was deposited by spin-coating a chlorobenzene solution of PYD2 mixed with **2** or **3** at various doping concentrations. The device characteristics are provided in Figures S6 and S7 and Tables S5 and S6 (Supplementary Materials). A comparison of the best device performances for **2** and **3** is also shown in Figure 5c,d. At all doping concentrations, the devices doped with **2** exhibit orange EL (Figure S6). The emission with a peak wavelength around 560 nm corresponds to the Commission international de l’éclairage (CIE) coordinates (0.43, 0.54). The turn-on voltage (V_on_) (brightness of 1 cd/m^2^) was found to almost insensitive to the dopant concentration, being 6.2, 6.3, 6.4 and 6.2 V for the devices with 8, 16, 24 and 40 wt% doping levels of **2**. This feature is different from the devices doped with the Ag(I) counterparts bearing the same ligands. The highest device efficiencies were recorded at the doping level of 24 wt% for which a peak current efficiency of 18.23 cd/A, a maximum power efficiency of 9.98 lm/W, and a maximum EQE of 5.91% were achieved. In agreement with their PL differences, complex **3** exhibits red-shifted electroluminescence compared with **2** and the CIE is close to (0.51, 0.49) (Figure S7 and Figure 5c). Notably, the maximum EQE of the device containing **3** is 7.96%, higher than that for **2**. In view of their PLQYs (0.71 for **2** vs. 0.48 for **3**), a much higher exciton utilization efficiency is estimated for **3** than for **2**, presumably due to the significantly shorter emission lifetime for **3**.

## 3. Materials and Methods

### 3.1. General Information

All Chemicals were purchased from commercial sources and used as received. The solvents for reactions were purified by solvent purification system before use. ^1^H and ^31^P{^1^H} nuclear magnetic resonance (NMR) spectra were recorded on an AVANCE 400 MHz superconducting-magnet high-field NMR spectrometer (Bruker, Zürich, Switzerland). Elemental analyses (C, H, N) were carried using a Vario EL III elemental analyzer (Elementar, Berlin, Germany). Thermogravimetric analysis (TGA) was performed on a Discovery TGA 55 instrument (TA Instruments, New Castle, DE, USA). UV-Vis absorption spectra were recorded on a UV-2600 spectrophotometer (Shimadzu, Japan). Photoluminescence (PL) spectra were recorded on a F-7100 fluorescence spectrophotometer (Hitachi, Tokyo, Japan). The transient PL decay characteristics were measured by a FluoTime 300 spectrometer (PicoQuant, Berlin, Germany). The variable–temperature transient PL measurements were performed with the aid of a continuous flow cryostat (Oxford Instruments, Ambingdon, UK). Absolute PLQYs were obtained using a Quantaurus-QY measurement system C13534 (Hamamatsu Photonics, Hamamatsu, Japan). Thin films for spectroscopic measurements were prepared by spin-coating a dichloromethane (DCM) solution of the host and the complex followed by thermal annealing at 60 °C for 0.5 h. Cyclic voltammetry was measured in nitrogen-purged DCM by a CHI600 electrochemical analyzer (Chenhua, China) with a glassy carbon working electrode, a platinum wire counter electrode, and an Ag/AgCl reference electrode. Tetrabutylammonium hexafluorophosphate (TBAPF_6_) (0.1 M) was used as the supporting electrolyte and ferrocenium/ferrocene (Fc^+^/Fc) was used as the internal standard.

### 3.2. General Procedure for the Synthesis of Cu(I) Complexes

A mixture of (Cu(CH_3_CN)_4_)BF_4_ (1.0 mmol) and diimine ligand (1.0 mmol) in acetonitrile/CH_2_Cl_2_ (10 mL/10mL) was stirred at room temperature for 1h and then added corresponding POP or Xantphos (1.0 mmol) in CH_2_Cl_2_ (10 mL). The reaction mixture was stirred for another 1 h, and the solvent was then evaporated. Single crystals of these complexes suitable for X-ray diffraction measurements were obtained through slow evaporation of their solutions in mixed hexane/dichloromethane (*v*/*v*, 3:1).

**(Cu(DMAC-PyPI)(POP))BF_4_ (1).** Yield: 81%. ^1^H NMR (400 MHz, CDCl_3_, 298 K) δ 9.15 (d, *J* = 7.9 Hz, 1H), 8.71 (d, *J* = 8.4 Hz, 1H), 8.59 (d, *J* = 8.4 Hz, 1H), 8.44 (s, 1H), 7.92 (d, *J* = 7.0 Hz, 3H), 7.60 (d, *J* = 7.9 Hz, 4H), 7.45 (d, *J* = 7.6 Hz, 3H), 7.30 (t, *J* = 7.8 Hz, 1H), 7.22 (t, *J* = 7.4 Hz, 2H), 7.16–7.08 (m, 10H), 7.05–6.96 (m, 20H), 6.77 (d, *J* = 8.1 Hz, 2H), 6.65 (d, *J* = 8.9 Hz, 1H), 6.29 (d, *J* = 8.0 Hz, 2H), 1.46 (s, 6H). ^31^P NMR (162 MHz, CDCl_3_, 298 K) δ −11.74 (s). Anal. calcd for C_77_H_58_BCuF_4_N_4_OP_2_.0.5CH_2_Cl_2_: C, 71.05; H, 4.54; N, 4.28. Found: C, 70.92; H, 4.84; N, 4.15.

**(Cu(DMAC-PyPI)(Xantphos))BF_4_ (2).** Yield: 79%. ^1^H NMR (400 MHz, CDCl_3_, 298 K) δ 8.85 (d, *J* = 7.7 Hz, 1H), 8.71 (d, *J* = 8.4 Hz, 1H), 8.60 (d, *J* = 8.5 Hz, 1H), 7.95 (d, *J* = 7.2 Hz, 3H), 7.84 (s, 1H), 7.62 (m, 4H), 7.46 (d, *J* = 7.8 Hz, 5H), 7.33 (m, 3H), 7.18 (t, *J* = 7.2 Hz, 2H), 7.10 (m, 5H), 7.06–6.87 (m, 18H), 6.77–6.71 (m, 2H), 6.60–6.55 (m, 2H), 5.81 (d, *J* = 8.1 Hz, 2H), 1.63 (s, 6H), 1.61 (s, 6H). ^31^P NMR (162 MHz, CDCl_3_, 298 K) δ −12.77 (s). Anal. calcd for C_80_H_62_BCuF_4_N_4_OP_2_.0.5CH_2_Cl_2_.EtOH: C, 70.97; H, 4.98; N, 4.01. Found: C, 71.01; H, 4.87; N, 4.01.

**(Cu(PXZ-PyPI)(POP))BF_4_ (3).** Yield: 84%. ^1^H NMR (400 MHz, CDCl_3_, 298 K) δ 9.13 (d, *J* = 7.9 Hz, 1H), 8.71 (d, *J* = 8.4 Hz, 1H), 8.58 (d, *J* = 8.4 Hz, 1H), 8.33 (d, *J* = 2.0 Hz, 1H), 7.97–7.90 (m, 3H), 7.61 (m, 4H), 7.47 (t, *J* = 7.7 Hz, 1H), 7.33–7.29 (m, 1H), 7.25–7.19 (m, 4H), 7.18–6.94 (m, 26H), 6.79 (m, 5H), 6.54 (dt, *J* = 8.6, 4.5 Hz, 2H), 5.65 (d, *J* = 8.0 Hz, 2H). ^31^P NMR (162 MHz, CDCl_3_, 298 K) δ −12.30 (s). Anal. calcd for C_74_H_52_BCuF_4_N_4_O_2_P_2_. 0.5CH_2_Cl_2_: C, 69.69; H, 4.16; N, 4.36. Found: C, 70.16; H, 4.32; N, 4.17.

**(Cu(PXZ-PIPy)(Xantphos))BF_4_ (4).** Yield: 77%. ^1^H NMR (400 MHz, CDCl_3_, 298 K) 9.22 (d, *J* = 7.4 Hz, 1H), 8.72 (d, *J* = 8.3 Hz, 1H), 8.64 (d, *J* = 8.4 Hz, 1H), 8.00–7.93 (m, 3H), 7.73 (dd, *J* = 6.6, 2.9 Hz, 2H), 7.64–7.52 (m, 3H), 7.41–7.30 (m, 4H), 7.25–7.09 (m, 14H), 7.05 (t, *J* = 7.9 Hz, 5H), 6.93–6.80 (m, 6H), 6.73–6.65 (m, 4H), 6.47 (dt, *J* = 7.6, 3.9 Hz, 2H), 6.36 (t, *J* = 7.6 Hz, 2H), 5.30 (d, *J* = 8.0 Hz, 2H), 1.60 (s, 6H). ^31^P NMR (162 MHz, CDCl_3_, 298 K) δ −11.83 (s). Anal. calcd for C_77_H_56_BCuF_4_N_4_O_2_P_2_: C, 72.16; H, 4.40; N, 4.37. Found: C, 71.93; H, 4.17; N, 4.06.

## 4. Conclusions

A new series of Cu(I) complexes exhibiting green-orange TADF emissions have been developed by using simple D-A type N^N ligands and ancillary phosphine ligands. The metal ion coordination induces a decrease of Δ*E*_ST_ for the ligand-centered excited states, resulting in the emission switching from fluorescence for free N^N ligands to TADF for the Cu(I) complexes. High PLQYs of up to 0.71 in doped films have been recorded for the Cu(I) complexes. The donor strength of the N^N ligand has a large impact on the energy level of the intraligand charge-transfer excited state and thus on the energetic ordering of the ^3^CT and ^3^LE states. As a result, the emission lifetimes of the Cu(I) complexes vary by more than 4-folds. High efficiency OLEDs with maximum EQEs of up to 7.96% have been achieved and the shorter emission lifetime was found to be beneficial for a high EQE. The encouraging results combined with rational design strategies in this study may provide a guide for developing highly efficient cuprous complexes and OLEDs.

## Figures and Tables

**Table 1:** The photophysical properties of complexes **1**–**4**.

Complex	In DCM		In DPEPO				
	λ_em_ [nm] ^1^	PLQY ^1,2^	λ_em_ [nm] ^3^	λ_em_ [nm] ^4^	S_1_/T_1_/Δ*E*_ST_^5^ [eV]	*τ*/[μs] ^6^	PLQY ^2,3^
**1**	514	0.06	534	544	2.56/2.45/0.11	21.4	0.62
**2**	518	0.095	533	545	2.55/2.47/0.08	24.1	0.71
**3**	534	0.05	565	582	2.46/2.39/0.07	5.8	0.48
**4**	537	0.042	564	575	2.48/2.43/0.05	4.3	0.42

^1^ Measured in degassed dichloromethane (DCM) solution (1 × 10^−5^ M) at 298 K. ^2^ Absolute photoluminescence quantum yields (PLQY) with an error of (±10%). ^3^ Measured in 10 wt.% doped DPEPO film 298 K. ^4^ Measured in DPEPO film at 77 K. ^5^ Estimated from the emission onsets of the fluorescence (298 K) and phosphorescence (77 K) spectra in the DPEPO film. ^6^ Emission decay times of emitters doped into DPEPO.

## Data Availability

The data presented in this study are available in article and Supplementary Materials here.

## Supplementary Materials

The following are available online: computational methods; details for device fabrications and measurements; TGA curves of 1–4 (Figure S1); crystal data and selected structural parameters of 1–4 (Tables S1 and S2); calculated FMOs of 1 and 3 (Figure S2); emission spectra and decay characteristics of 1–4 in PMMA and mCP films (Figures S3 and S4 and Table S3); cyclic voltammograms of 1–4 (Figure S5 and Table S4); device performances of 2 and 3 (Figures S6 and S7 and Tables S5 and S6).

## Sample Availability

Samples of the compounds are not available from the authors.

## References

[1] Li K., Tong G.S.M., Wan Q., Cheng G., Tong W.-Y., Ang W.-H., Kwong W.-L., Che C.-M. (2016). Highly phosphorescent platinum(II) emitters: Photophysics, materials and biological applications. Chem. Sci..

[2] Yang X., Zhou G., Wong W.-Y. (2015). Functionalization of phosphorescent emitters and their host materials by main-group elements for phosphorescent organic light-emitting devices. Chem. Soc. Rev..

[3] Fan C., Yang C. (2014). Yellow/orange emissive heavy-metal complexes as phosphors in monochromatic and white organic light-emitting devices. Chem. Soc. Rev..

[4] Xu H., Chen R., Sun Q., Lai W., Su Q., Huang W., Liu X. (2014). Recent progress in metal–organic complexes for optoelectronic applications. Chem. Soc. Rev..

[5] Chou P.-T., Chi Y., Chung M.-W., Lin C.-C. (2011). Harvesting luminescence via harnessing the photophysical properties of transition metal complexes. Coord. Chem. Rev..

[6] Evans R.C., Douglas P., Winscom C.J. (2006). Coordination complexes exhibiting room-temperature phosphorescence: Evaluation of their suitability as triplet emitters in organic light emitting diodes. Coord. Chem. Rev..

[7] Ho P.-Y., Ho C.-L., Wong W.-Y. (2020). Recent advances of iridium(III) metallophosphors for health-related applications. Coord. Chem. Rev..

[8] Zhang X., Hou Y., Xiao X., Chen X., Hu M., Geng X., Wang Z., Zhao J. (2020). Recent development of the transition metal complexes showing strong absorption of visible light and long-lived triplet excited state: From molecular structure design to photophysical properties and applications. Coord. Chem. Rev..

[9] Arias-Rotondo D.M., McCusker J.K. (2016). The photophysics of photoredox catalysis: A roadmap for catalyst design. Chem. Soc. Rev..

[10] Zhao J., Wu W., Sun J., Guo S. (2013). Triplet photosensitizers: From molecular design to applications. Chem. Soc. Rev..

[11] Barbieri A., Accorsi G., Armaroli N. (2008). Luminescent complexes beyond the platinum group: The d^10^ avenue. Chem. Commun..

[12] Wenger O.S. (2018). Photoactive Complexes with Earth-Abundant Metals. J. Am. Chem. Soc..

[13] To W.-P., Chan K.T., Tong G.S.M., Ma C., Kwok W.-M., Guan X., Low K.-H., Che C.-M. (2013). Strongly luminescent gold(III) complexes with long-lived excited states: High emission quantum yields, energy up-conversion, and nonlinear optical properties. Angew. Chem. Int. Ed..

[14] To W.-P., Tong G.S.M., Lu W., Ma C., Liu J., Chow A.L.-F., Che C.-M. (2012). Luminescent organogold(III) complexes with long-lived triplet excited states for light-induced oxidative C–H bond functionalization and hydrogen production. Angew. Chem. Int. Ed..

[15] Uoyama H., Goushi K., Shizu K., Nomura H., Adachi C. (2012). Highly efficient organic light-emitting diodes from delayed fluorescence. Nature.

[16] Czerwieniec R., Yu J., Yersin H. (2011). Blue-light emission of Cu(I) complexes and singlet harvesting. Inorg. Chem..

[17] Chen X.-L., Yu R., Zhang Q.-K., Zhou L.-J., Wu X.-Y., Zhang Q., Lu C.-Z. (2013). Rational design of strongly blue-emitting cuprous complexes with thermally activated delayed fluorescence and application in solution-processed OLEDs. Chem. Mater..

[18] Chen X.-L., Lin C.-S., Wu X.-Y., Yu R., Teng T., Zhang Q.-K., Zhang Q., Yang W.-B., Lu C.-Z. (2015). Highly efficient cuprous complexes with thermally activated delayed fluorescence and simplified solution process OLEDs using the ligand as host. J. Mater. Chem. C.

[19] Liang D., Chen X.-L., Liao J.-Z., Hu J.-Y., Jia J.-H., Lu C.-Z. (2016). Highly efficient cuprous complexes with thermally activated delayed fluorescence for solution-processed organic light-emitting devices. Inorg. Chem..

[20] Klein M., Rau N., Wende M., Sundermeyer J., Cheng G., Che C.-M., Schinabeck A., Yersin H. (2020). Cu(I) and Ag(I) complexes with a new type of rigid tridentate n,p,p-ligand for thermally activated delayed fluorescence and OLEDs with high external quantum efficiency. Chem. Mater..

[21] So G.K.-M., Cheng G., Wang J., Chang X., Kwok C.-C., Zhang H., Che C.-M. (2017). Efficient color-tunable copper(I) complexes and their applications in solution-processed organic light-emitting diodes. Chem. Asian J..

[22] Osawa M., Hoshino M., Hashimoto M., Kawata I., Igawa S., Yashima M. (2015). Application of three-coordinate copper(I) complexes with halide ligands in organic light-emitting diodes that exhibit delayed fluorescence. Dalton Trans..

[23] Czerwieniec R., Leitl M.J., Homeier H.H.H., Yersin H. (2016). Cu(I) complexes—Thermally activated delayed fluorescence. Photophysical approach and material design. Coord. Chem. Rev..

[24] To W.-P., Cheng G., Tong G.S.M., Zhou D., Che C.-M. (2020). Recent advances in metal-TADF emitters and their application in organic light-emitting diodes. Front. Chem..

[25] Mahoro G.U., Fernandez-Cestau J., Renaud J.-L., Coto P.B., Costa R.D., Gaillard S. (2020). Recent advances in solid-state lighting devices using transition metal complexes exhibiting thermally activated delayed fluorescent emission mechanism. Adv. Optical Mater..

[26] Shi S., Jung M.C., Coburn C., Tadle A., Sylvinson M.R.D., Djurovich P.I., Forrest S.R., Thompson M.E. (2019). Highly efficient photo- and electroluminescence from two-coordinate Cu(I) complexes featuring nonconventional N-heterocyclic carbenes. J. Am. Chem. Soc..

[27] Hamze R., Peltier J.L., Sylvinson D., Jung M., Cardenas J., Haiges R., Soleilhavoup M., Jazzar R., Djurovich P.I., Bertrand G. (2019). Eliminating nonradiative decay in Cu(I) emitters: >99% quantum efficiency and microsecond lifetime. Science.

[28] Zhu Z.-Q., Fleetham T., Turner E., Li J. (2015). Harvesting all electrogenerated excitons through metal assisted delayed fluorescent materials. Adv. Mater..

[29] Chan K.-T., Lam T.-L., Yu D., Du L., Phillips D.L., Kwong C.-L., Tong G.S.-M., Cheng G., Che C.-M. (2019). Strongly luminescent tungsten emitters with emission quantum yields up to 84%: TADF and high-efficiency molecular tungsten OLEDs. Angew. Chem. Int. Ed..

[30] Zhou D., To W.-P., Tong G.S.M., Cheng G., Du L., Phillips D.L., Che C.-M. (2020). Tetradentate gold(III) Complexes as thermally activated delayed fluorescence (TADF) emitters: Microwave-assisted synthesis and high-performance OLEDs with long operational lifetime. Angew. Chem. Int. Ed..

[31] Teng T., Li K., Cheng G., Wang Y., Wang J., Li J., Zhou C., Liu H., Zou T., Xiong J. (2020). Lighting silver(I) complexes for solution-processed organic light-emitting diodes and biological applications via thermally activated delayed fluorescence. Inorg. Chem..

[32] Jia J.-H., Liang D., Yu R., Chen X.-L., Meng L., Chang J.-F., Liao J.-Z., Yang M., Li X.-N., Lu C.-Z. (2020). Coordination-induced thermally activated delayed fluorescence: From non-TADF donor–acceptor-type ligand to TADF-active Ag-based complexes. Chem. Mater..

[33] Zhang Y., Schulz M., Wächtler M., Karnahl M., Dietzek B. (2018). Heteroleptic diimine–diphosphine Cu(I) complexes as an alternative towards noble-metal based photosensitizers: Design strategies, photophysical properties and perspective applications. Coord. Chem. Rev..

[34] Mara M.W., Fransted K.A., Chen L.X. (2015). Interplays of excited state structures and dynamics in copper(I) diimine complexes: Implications and perspectives. Coord. Chem. Rev..

[35] Yersin H., Rausch A.F., Czerwieniec R., Hofbeck T., Fischer T. (2011). The triplet state of organo-transition metal compounds. Triplet harvesting and singlet harvesting for efficient OLEDs. Coord. Chem. Rev..

[36] Ravinson D.S.M., Thompson M.E. (2020). Thermally assisted delayed fluorescence (TADF): Fluorescence delayed is fluorescence denied. Mater. Horiz..

