# Supplementary material for: Solution-Processed OLEDs Based on Thermally Activated Delayed Fluorescence Copper(I) Complexes with Intraligand Charge-Transfer Excited State
Source: Molecules. 2021 Feb 20;26(4):1125. doi: 10.3390/molecules26041125 (PMC7924317; doi:10.3390/molecules26041125)
Supplement: Supplementary file 1 [file molecules-26-01125-s001.pdf]

Supplementary Materials

Solution-Processed OLEDs Based on Thermally Activated Delayed Fluorescence Copper(I) Complexes with Intraligand Charge-Transfer Excited State

Teng Teng[1,2,†], Jinfan Xiong[1,†], Gang Cheng[3], Changjiang Zhou[1,2], Xialei Lv[1,2] and Kai Li[1,*]

[1] College of Materials Science and Engineering, Shenzhen University, Shenzhen, 518055, China

[2] College of Physics and Optoelectronic Engineering, Shenzhen University, Shenzhen 518060, China

[3] State Key Laboratory of Synthetic Chemistry, HKU-CAS Joint Laboratory on New Materials, and Department of Chemistry, The University of Hong Kong, Pokfulam Road, Hong Kong, China

[†] These authors contributed equally to this work.

Corresponding Author:

*E-mail: kaili@szu.edu.cn

Computational Methods

All the electronic structure calculations were carried out with Gaussian 09 (version D.01) package. The geometries of molecules were achieved form single-crystal X-ray diffraction analysis. The frontier molecular orbital properties were obtained by DFT method at PBE0 functional in conjunction with the polarizable continuum model (PCM) using dichloromethane as the solvent. The relativistic effective core potential and SDD basis were employed for Ag(I) atoms, and an all-electron basis set of 6-31G* for all other atoms.

Device Fabrication and Measurement

Poly(3,4-ethylenedioxythiophene):poly(styrene sulfonic acid) (PEDOT:PSS) was purchased from Heraeus. N,N′-bis(4-(6-((3-ethyloxetan-3-yl) methoxy))-hexylphenyl)-N,N′-diphenyl-4,4′-diamine (OTPD), di(9H-carbazol-9-yl)pyridine (PYD2), bis[2-(diphenylphosphino)phenyl]ether oxide (DPEPO), and 1,3,5-tris(1-phenyl-1H-benzo[d]imidazol-2-yl)benzene (TPBi) were purchased from Luminescence Technology Corp. All of these materials were used as received. The device structure was ITO/PEDOT:PSS/OTPD/PYD2:emitter/DPEPO/TPBi/LiF/Al. An aqueous solution of PEDOT:PSS was spin-coated onto the cleaned ITO coated glass substrate and baked at 120 ^{o}C for 20 min to remove the residual water solvent in a clean room. The crosslinkable OTPD in toluene was spin-casted on top of the PEDOT:PSS layer and heated at 200 ℃ for 30 min to carry out crosslinking inside a N_2-filled glove box. The crosslinked OTPD was then subjected to spin chlorobenzene solvent for three times to remove the unreacted moieties. Afterwards, the mixture of PYD2 and Cu(I) complex in chlorobenzene was spin-coated atop the OTPD layer inside the glove box. After annealed at 70 ℃ for 30 min, all devices were subsequently transferred into a Kurt J. Lesker SPECTROS vacuum deposition system without exposing to air. In the vacuum chamber, organic materials of DPEPO and TPBi were thermally deposited in sequence at a rate of ~0.1 nm s^{-1}. Finally, LiF (1.2 nm) and Al (100 nm) were thermally deposited at rates of 0.03 and 0.2 nm s^{-1}, respectively. Luminance-current-voltage characteristics, CIE coordinates, EL spectra, current efficiency, power efficiency and EQE were measured using a Keithley 2400 source-meter and an absolute external quantum efficiency measurement system (C9920-12, Hamamatsu Photonics). All devices were characterized at room temperature in air without encapsulation.

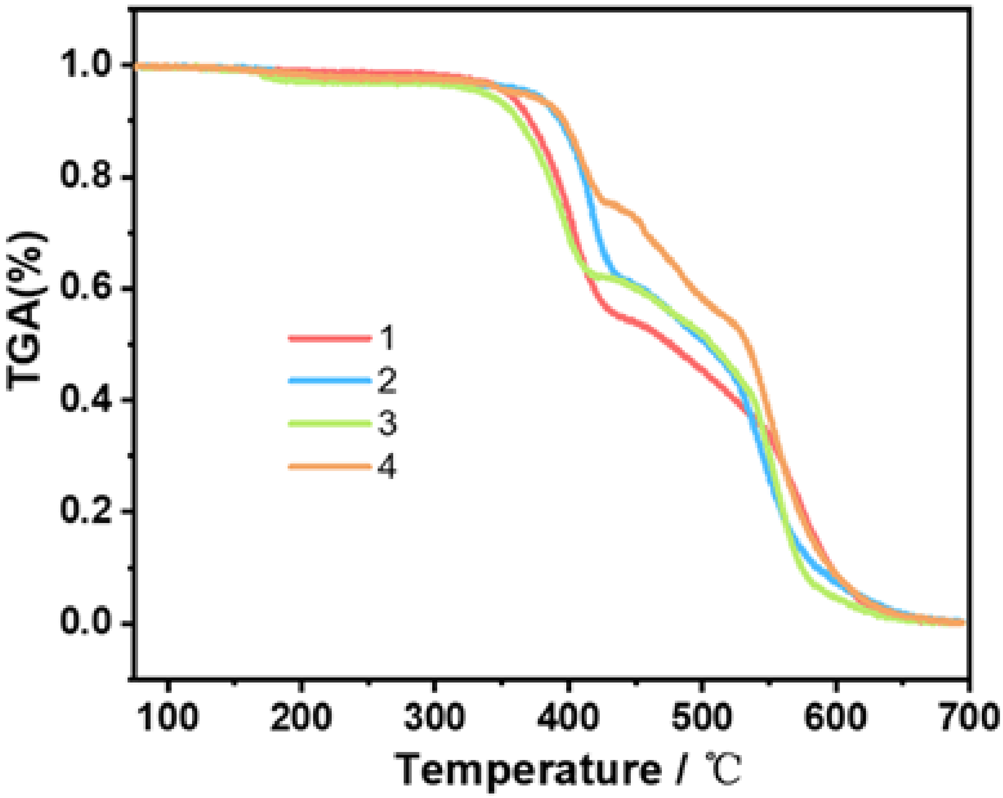

Figure S1. Thermal gravimetric analysis (TGA) curves of complexes **1**, **2**, **3** and **4** under N_2 atmosphere with a heating ramp of 10 ^{o}C/min.

Table S1. Crystal data

Compound	**1**	**2**	**3**	**4**
CCDC NO.	2022615	2022616	2022617	2022618
Formula	$C_{154}H_{116}BCu_2F_4N_8O_2P_4$	$C_{80}H_{62}BCuF_4N_4OP_2$	$C_{75}H_{54}BCl_2CuF_4N_4O_2P_2$	$C_{77}H_{56}CuN_4O_2P_2$
Formula weight	2448.31	1307.62	1326.41	1194.73
Crystal system	monoclinic	monoclinic	monoclinic	monoclinic
Space group	C2/c	$P2_1/c$	Pn	$P2_1/n$
a/Å	34.025(5)	12.8293(15)	14.6141(11)	13.6061(5)
b/Å	22.288(4)	18.830(2)	9.5312(7)	26.3164(11)
c/Å	21.469(7)	30.264(3)	22.5165(18)	20.4803(7)
α/°	90	90	90	90.00
β/°	127.723(3)	95.695(3)	93.001(2)	105.5250(10)
γ/°	90	90	90	90
Volume/Å^3	12878(5)	7274.8(14)	3132.0(4)	7065.7(5)
Z	4	4	2	4
ρ_{calc}g/cm^3	1.263	1.194	1.406	1.123
μ/mm^{-1}	0.443	0.400	0.550	0.400
F(000)	5084.0	2712.0	1364.0	2484.0
Crystal size/mm^3	0.05 × 0.04 × 0.01	0.15 × 0.08 × 0.05	0.16 × 0.05 × 0.03	0.15 × 0.05 × 0.03
Θ range (deg)	3.978-50.054	3.854-52.788	4.274-52.822	4.164-52.774
Reflections collected/unique	43960/11299	58755/14812	28744 / 11199	53953 /14032
GOF on F^2	1.000	1.014	1.032	1.027
R_1/wR_2 [I>=2σ (I)]	0.0634/ 0.1372	0.0593/ 0.1263	0.0522/ 0.0887	0.0561/ 0.1293
R_1/wR_2 [all data]	0.1561/0.1785	0.1322/0.1595	0.1004/ 0.1081	0.1000/ 0.1582

Table S2. Selected bond length (Å) and bond angles (deg) for complexes **1**, **2**, **3**, and **4**

	**1**	**2**	**3**	**4**
Bond length (Å)				
**Cu-P1**	2.2941(15)	2.2608(11)	2.281(2)	2.2511(10)
**Cu-P2**	2.2377(15)	2.2430(11)	2.218(2)	2.2696(10)
**Cu-N1**	2.104(4)	2.139(3)	2.086(5)	2.113(3)
**Cu-N2**	2.082(4)	2.056(3)	2.069(6)	2.061(3)
Angles (degree)				
**N1-Cu-P1**	100.87(11)	107.68(9)	100.79(16)	112.35(8)
**N1-Cu-P2**	110.28(12)	106.52(9)	122.11(17)	100.98(8)
**N2-Cu-P1**	102.42(11)	113.25(9)	102.18(17)	121.39(8)
**N2-Cu-P2**	136.88(11)	125.54(9)	125.87(16)	116.78(8)
**N1-Cu-N2**	78.10(16)	79.98(11)	79.6(2)	79.52(11)
**P1-Cu-P2**	116.38(5)	115.41(4)	118.20(8)	116.38(4)
Dihedral angles (degree)				
**N1-Cu-N2/P1-Cu-P2**	85.877	85.855	89.842	85.299

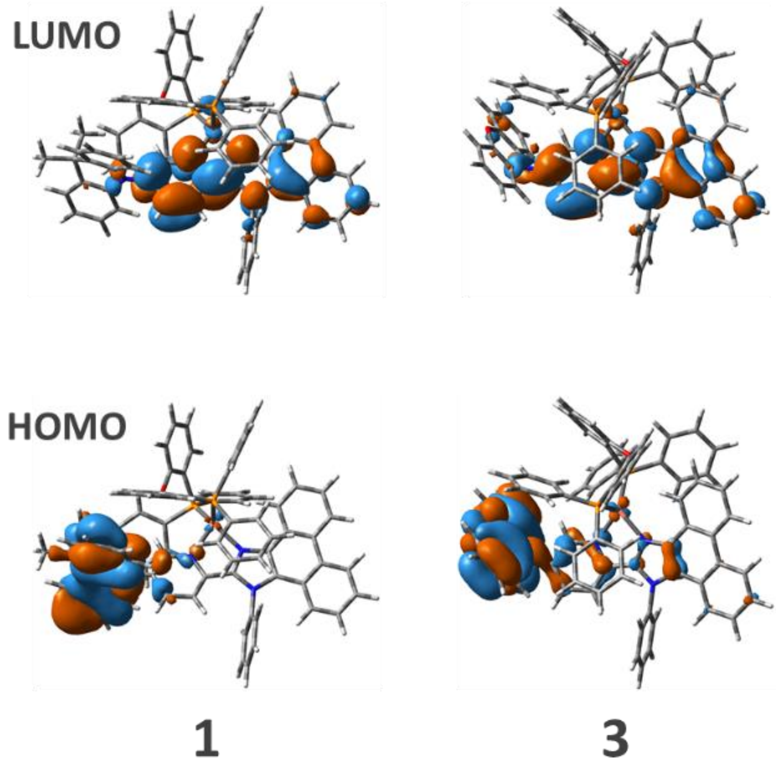

Figure S2. Frontier molecular orbitals for complexes **1** and **3**.

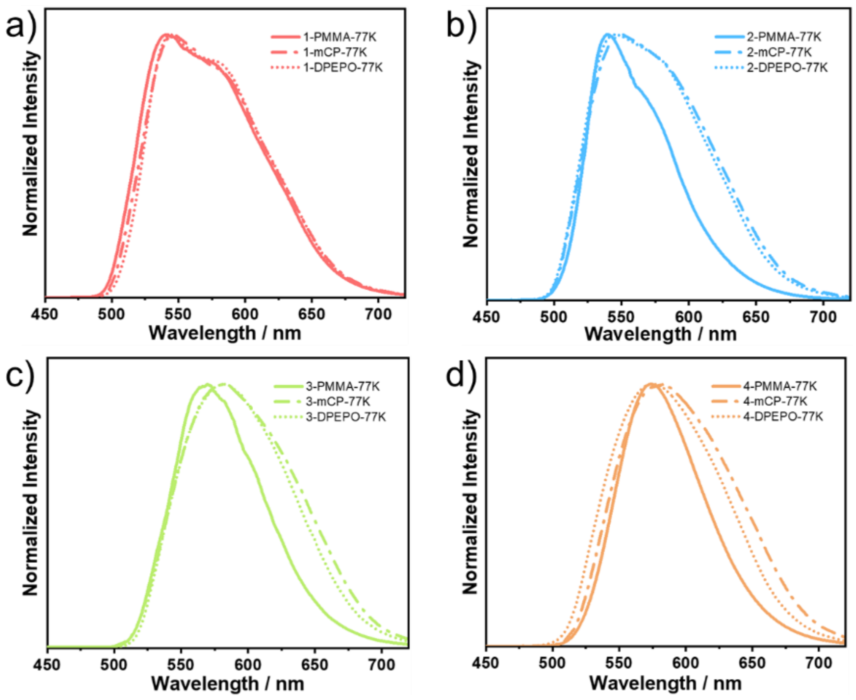

Figure S3. Phosphorescence spectra in 77 K for the doped films in PMMA, mCP and DPEPO host for (a) **1**; (b) **2**; (c) **3**; (d) **4**.

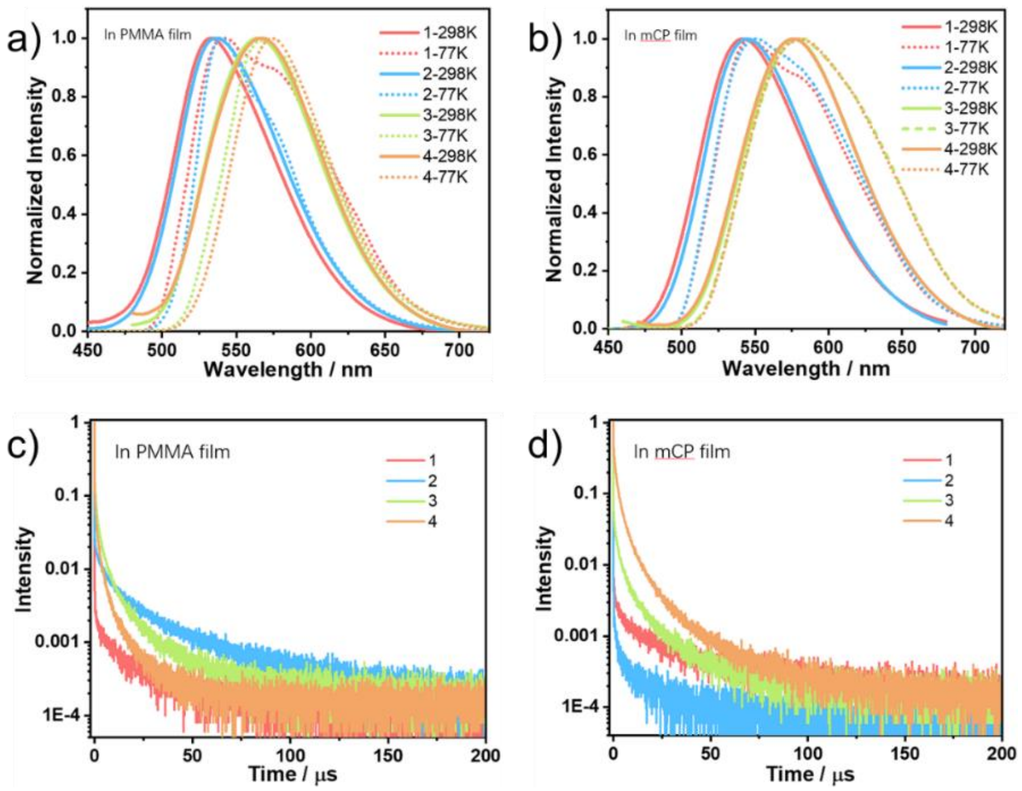

Figure S4. a) Fluorescence (298 K) and phosphorescence (77K) of 10 wt% emitters doped into the PMMA host. b) Fluorescence (298 K) and phosphorescence (77K) of 10 wt% emitters doped into the mCP host. c) transient PL decay characteristics for **1**, **2**, **3** and **4** doped into PMMA films (10 wt.%) at 298K. d) transient PL decay characteristics for **1**, **2**, **3** and **4** doped into mCP films (10 wt.%) at 298K.

Table S3. The physical properties and kinetic parameters of complexes 1–4

Complex	λ_{em}[nm][a]	τ/[μs][b]	λ_{em}[nm][c]	τ/[μs][d]
**1**	533/542	22.9	541/547	28.2
**2**	535/540	21.9	543/551	15.7
**3**	563/570	7.6	575/583	8.6
**4**	566/575	4.2	576/582	3.2

[a]Measured in PMMA film at 298/77 K. [b] Measured in PMMA film at 298 K. [c]Measured in mCP film at 298/77 K. [d] Measured in mCP film at 298 K.

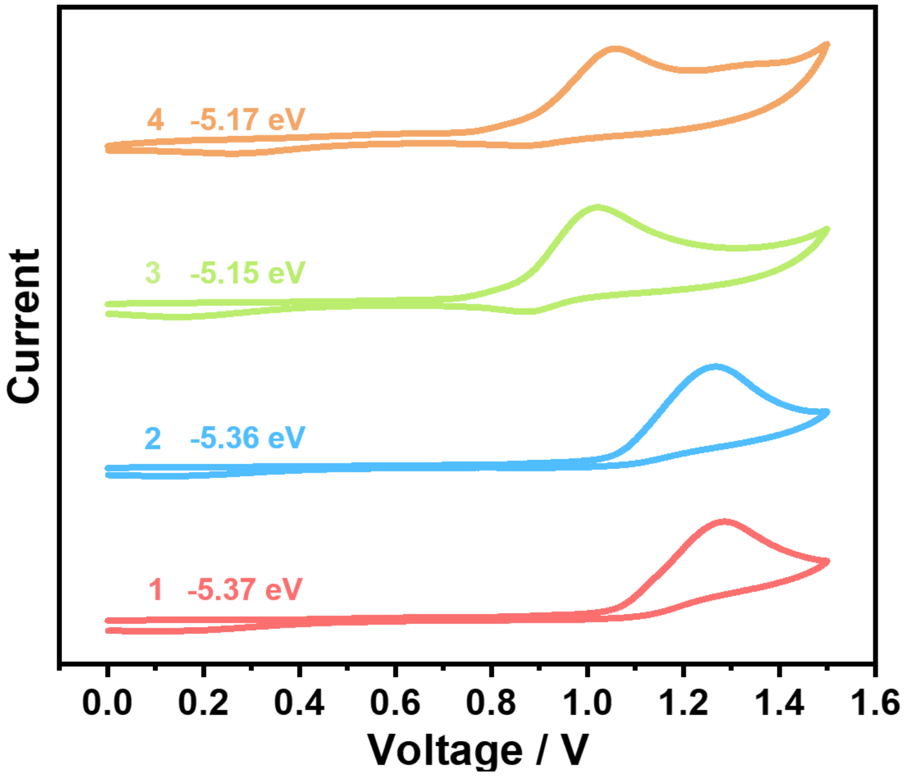

Figure S5. Cyclic voltammograms of **1**, **2**, **3** and **4** in film with an Ag wire pseudo-reference electrode. ferrocene as internal standard.

Table S4. HOMO and LUMO energy levels of **1–4** determined by cyclic voltammograms and optical gaps

Complex	E_{HOMO}[a]	E_g [eV][b]	E_{LUMO}[c]
**1**	-5.37	2.58	-2.79
**2**	-5.36	2.58	-2.78
**3**	-5.15	2.36	-2.79
**4**	-5.17	2.36	-2.81

[a]The HOMO energy level of the compound was calculated using the formula: E_{HOMO} (eV) = -[4.8 + E_{ox} - $E_{1/2}(Fc^+/Fc)$)] eV. [b]Optical bandgap determined from the absorption onset. [c]The LUMO energy levels of the compound can be deduced from the HOMO level and optical bandgap: E_{LUMO} (eV) = (E_{HOMO} + E_g) eV.

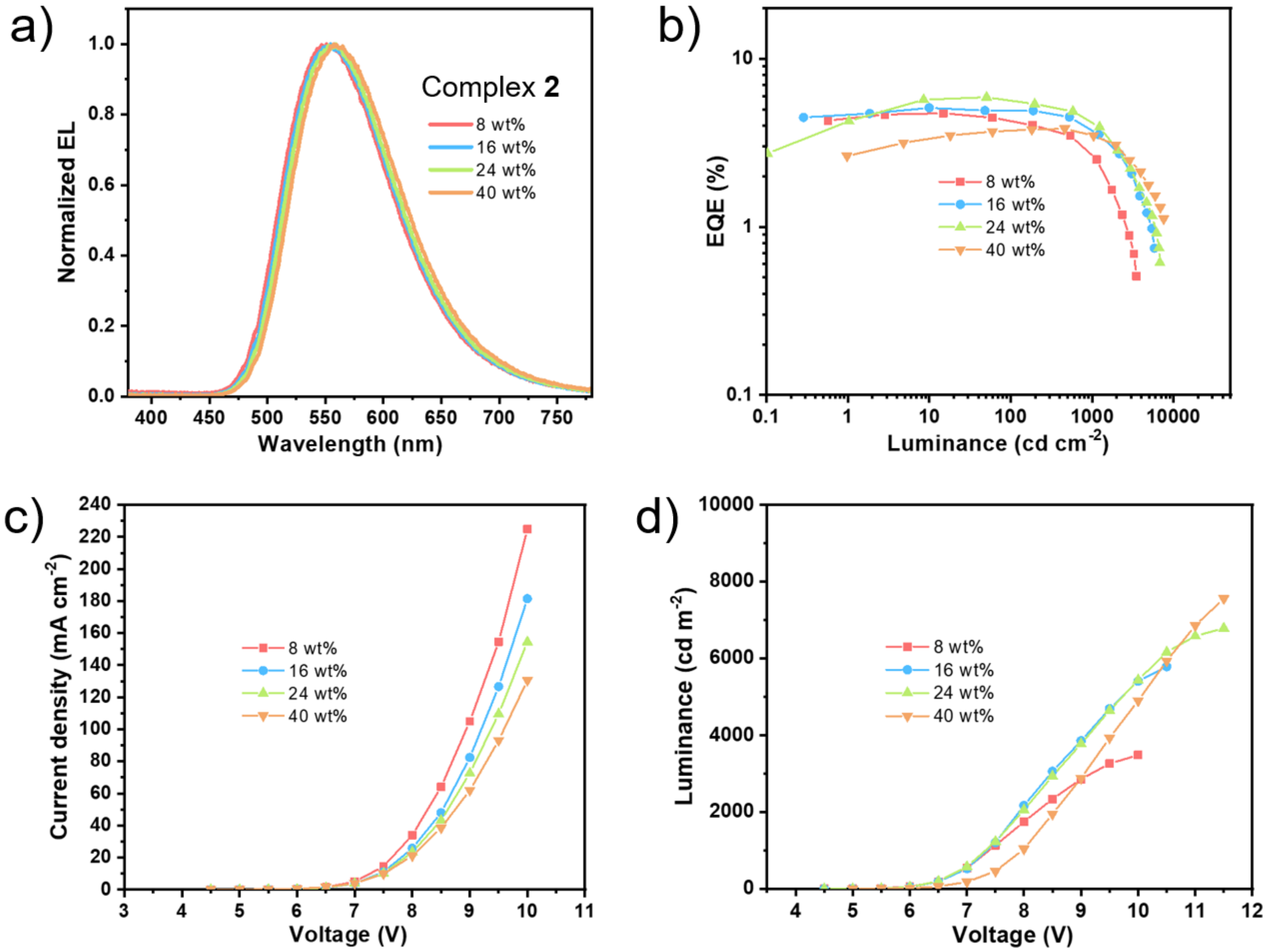

Figure S6. a) EL spectra of devices. b) plots of external quantum efficiency versus luminance. c) current density-voltage (I-V) and d) luminance-voltage characteristics of the devices.

Table S5. Summary of the OLED device data using **2** as the emitter

Concentration	V_{on} [V]	L [cd m^{-2}]	CE [cd A^{-1}]	PE [lmW^{-1}]	EQE %	CIE [(x, y)]
8 wt.%	6.2	3500	14.63	9.07	4.74	0.41, 0.55
16 wt.%	6.3	5790	15.64	9.64	5.11	0.42, 0.55
24 wt.%	6.4	6800	18.23	9.98	5.91	0.43, 0.54
40 wt.%	6.2	7560	11.63	5.54	3.84	0.44, 0.54

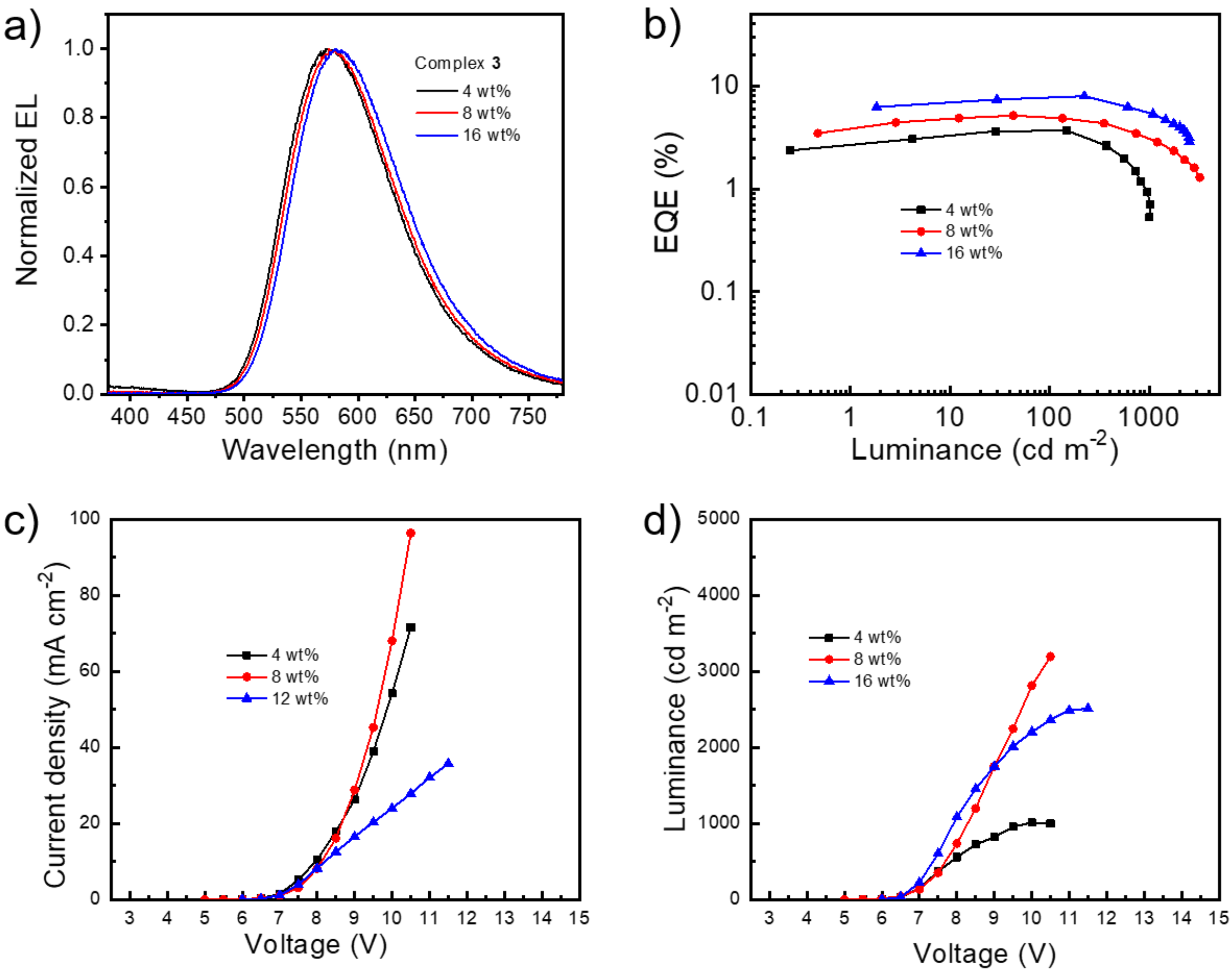

Figure S7. a) EL spectra of devices. b) plots of external quantum efficiency versus luminance. c) current density-voltage (I-V) and d) luminance-voltage characteristics of the devices.

Table S6. Summary of the OLED device data using **3** as the emitter

Concentration	L [cd m^{-2}]	CE [cd A^{-1}]	PE [lmW^{-1}]	EQE %	CIE [(x, y)]
4 wt.%	997	10.01	4.80	3.68	0.48, 0.50
8 wt.%	3200	13.68	6.69	5.16	0.49, 0.50
16 wt.%	2520	19.74	8.86	7.96	0.51, 0.49
